# NK Cells and PMN-MDSCs in the Graft From G-CSF Mobilized Haploidentical Donors Display Distinct Gene Expression Profiles From Those of the Non-Mobilized Counterpart

**DOI:** 10.3389/fimmu.2021.657329

**Published:** 2021-04-27

**Authors:** Andrea Pelosi, Francesca Besi, Nicola Tumino, Pietro Merli, Linda Quatrini, Giuseppina Li Pira, Mattia Algeri, Lorenzo Moretta, Paola Vacca

**Affiliations:** ^1^ Immunology Research Area, Bambino Gesù Children’s Hospital, IRCCS, Rome, Italy; ^2^ Department of Pediatric Hematology/Oncology, Bambino Gesù Children’s Hospital, IRCCS, Rome, Italy

**Keywords:** myeloid-derived suppressor cells, NK cells, hematopoietic stem cell transplantation (HSCT), leukemia, microarray gene expression analysis

## Abstract

A recent approach of hematopoietic stem cell (HSC) transplantation from haploidentical donors “mobilized” with G-CSF is based on the selective depletion of *αβ* T and B lymphocytes from the graft. Through this approach, the patient receives both HSC and mature donor-derived effector cells (including NK cells), which exert both anti-leukemia activity and protection against infections. We previously showed that donor HSC mobilization with G-CSF results in accumulation in the graft of polymorphonuclear myeloid-derived suppressor cells (PMN-MDSCs), capable of inhibiting *in vitro* the anti-leukemia activity of allogeneic NK cells. Here, we performed a detailed gene expression analysis on NK cells and PMN-MDSCs both derived from mobilized graft. Cytotoxicity assays and real time PCR arrays were performed in NK cells. Microarray technology followed by bioinformatics analysis was used for gene expression profiling in PMN-MDSCs. Results indicate that NK cells from the graft have a lower cytolytic activity as compared to those from non-mobilized samples. Further, mobilized PMN-MDSCs displayed a peculiar transcriptional profile distinguishing them from non-mobilized ones. Differential expression of pro-proliferative and immune-modulatory genes was detected in mobilized PMN-MDSCs. These data strengthen the concept that G-CSF-mobilized PMN-MDSCs present in the graft display unique molecular characteristics, in line with the strong inhibitory effect on donor NK cells.

## Introduction

Allogeneic hematopoietic stem cell transplantation (HSCT) is a life-saving treatment for children with both malignant and non-malignant severe disorders. However, a suitable HLA-compatible donor is not always available (1, 2). Thus, different HSCT strategies have been developed to offer an allograft to these patients with no alternative therapeutic options. The first approach was based on the infusion of megadoses of highly purified CD34^+^ precursors (3, 4). However, the complete lymphoid cell depletion caused a prolonged lymphopenia and a delayed immune reconstitution, resulting into an increased risk of leukemia relapses and opportunistic infections, especially in the early period after HSCT (5). In an attempt to overcome these severe complications, a novel method of graft manipulation based on the specific depletion of *αβ* T lymphocytes (responsible of graft-*versus*-host disease; GvHD) and B cells (in order to prevent Epstein–Barr virus-related post-transplant lymphoproliferative disorders), has been more recently introduced (6, 7). Through this new approach, the patient receives not only HSCs but also high numbers of cells of the innate immunity (including mature NK cells, *γδ* T lymphocytes and different myeloid cells), some of which may rapidly exert anti-leukemia activity (graft-*versus*-leukemia; GvL) and early protection against infections. In particular, infused mature donor NK cells can promptly exert a GvL effect (8–12).

To obtain high numbers of HSC in the peripheral blood (PB), donors were treated with G-CSF for 5 days. G-CSF induces HSC mobilization from the bone marrow (BM). However, this treatment also induces an increase of circulating myeloid cells, including myeloid-derived suppressor cells (MDSCs) (13–15).

MDSCs represent an intrinsic myeloid compartment derived from a common myeloid precursor present in the BM (16). Human MDSCs are classified in two major subsets based on their surface markers’ expression, namely monocytic MDSCs (Mo-MDSCs) and polymorphonuclear MDSCs (PMN-MDSCs) (17). Mo-MDSCs are CD45^+^Lin^−^(CD3/CD19/CD56)HLA-DR^−/low^CD33^+^CD11b^+^CD14^+^CD66b^−^ (or CD15^−^) while PMN-MDSCs are CD45^+^Lin^−^ (CD3/CD19/CD56)HLA-DR^−/low^CD33^+^CD11b^+^CD14^−^CD66b^+^ (or CD15^+^) (18). Low numbers of Mo- and PMN-MDSC cells are present in PB of healthy individuals. However, a substantial accumulation of these cell subsets both in PB and in tissues was detected in different pathological conditions (including acute/chronic viral and bacterial infections), characterized by an inflammatory response (19–22). As a consequence of inflammation, a partial block in the differentiation of myeloid precursors occurs contributing to their accumulation. Importantly, MDSCs have also been detected in the microenvironment of various tumors (22, 23).

Emerging evidence indicates that MDSCs can interact and regulate the function of other immune cells, including NK cells. Several studies described different mechanisms adopted by MDSCs to exert their immunomodulatory function either by mechanisms that require cell-to-cell contact and/or by the release of soluble factors (24). In addition, a large number of data indicate that some genes are critical for MDSC function and/or represent useful markers to identify PMN-MDSCs. Thus, lectin-type oxidized LDL receptor 1 (LOX1) is undetectable in neutrophils while it is expressed by PMN-MDSC with potent immunosuppressive activity (18). Arginase 1 (ARG1) and nitric oxide synthase 2 (NOS2) are enzymes involved in L-arginine metabolism and are expressed in activated MDSCs. ARG1 promotes depletion of L-arginine, whereas NOS2 stimulates nitric oxide synthesis. In an inflammatory/tumor microenvironment, both enzymes cause suppression of immune cell function (25). Another characteristic of MDSCs associated with their suppressive capability is the production of interleukin 10 (IL-10) and tumor growth factor β (TGF*β*), both acting as potent immunosuppressive cytokines on T and NK cell function (26). Furthermore, two important genes in MDSC biology are the S100 calcium-binding proteins S100A8 and S100A9. These proteins are constitutively expressed by myeloid cells including MDSCs, and they are down-regulated during normal differentiation of myeloid precursors. Some studies indicate that tumor-derived factors promote sustained up-regulation of S100A9 in myeloid precursors inducing accumulation of MDSCs (27).

In a previous study, we showed that, in HSCT, donor HSC mobilization with G-CSF induces the accumulation of PMN-MDSCs in PB and, consequently, in the graft and that these cells are capable of inhibiting *in vitro* the anti-leukemia cytolytic activity of donor-derived mature NK cells. Their potent immunosuppressive activity was exerted both by cell-to-cell contact mechanism and by their ability to release prostaglandin E2 (PGE2), indoleamine-pyrole 2, 3-dioxygenase (IDO) metabolites, and exosomes with immunosuppressive activity (28).

In the present study, we show that NK cells isolated from “mobilized” donors display a reduced cytolytic activity paralleled by a decreased expression of genes involved in this function as compared to non-mobilized NK cells. Thus, NK cell suppression detected in mobilized donors and the striking increase of PMN-MDSCs in the graft prompted us to explore more deeply the molecular features of these PMN-MDSCs. In particular, we evaluated the global pattern of gene expression in PMN-MDSCs present in the donor both before and after G-CSF-induced mobilization. Bioinformatics-based approaches were used to better investigate the role of these cells in HSCT. In mobilized PMN-MDSCs, we found de-regulation of some immune-modulatory genes and a striking activation of genes involved in the cell cycle program. These results show that mobilized PMN-MDSCs are characterized by a peculiar transcriptional profile compatible with an intense proliferative activity. Moreover, these data suggest of a further PMN-MDSC expansion in the patient after transplantation. Thus, removal or inactivation of this immunosuppressive cell subset could represent a promising strategy to restore/improve the NK-mediated GvL activity.

## Materials and Methods

### G-CSF Mobilized Donors, Samples, and Ethical Statements

Healthy donors were enrolled at Bambino Gesù Children’s Hospital, Rome, Italy. G-CSF mobilized donors received subcutaneous administration of G-CSF for five days (until apheresis) at the dose of 10^−^_12_ μg/kg/day. Peripheral blood mononuclear cells (PBMCs) were obtained from G-CSF mobilized healthy donors (n = 12) and non-mobilized healthy donors (HD; n = 9). PBMCs were obtained after density gradient centrifugation (Ficoll-Lympholyte, Cederlane) as described before. Both G-CSF mobilized donors and HD gave their informed consent to participate in this study, which was approved by the Bambino Gesù Children’s Hospital (Rome, Italy) ethics committees (Prot. n. 1724/2018) and was conducted in accordance with the tenets of the Declaration of Helsinki.

### Cell Isolation and Antibodies

NK cells and PMN-MDSCs were isolated from PBMCs of mobilized and non-mobilized donors using NK isolation kit and CD66b^+^ microbeads (purity >98%, data not shown) following manufacturer’s instruction (Miltenyi Biotec, Bergisch Gladbach, Germany). Before starting any experiment, we determined the purity of isolated cells by flow cytometry using anti-CD3-APC, anti-CD19-ECD, and CD56-PC7 (Beckman Coulter, Brea, CA) for NK cells. PMN-MDSCs were labeled with anti-CD3-AF700, anti-CD19-AF700, anti-CD11b-FITC, anti-CD33-PC7, anti-HLA-DR-PE, anti-CD14-ECD, anti-CD45-KrOr, and anti-CD66b-APC desiccated in the Duraclone custom design platform (Beckman Coulter, Brea, CA) adding anti-CD56-BV650 (BioLegend, San Diego, CA) and following manufacturer’s instruction. After the staining procedures, cells were acquired at Cytoflex LX and analyzed with Cytexpert software (v2.4, Beckman Coulter, Brea, CA). Freshly isolated NK cells were immediately used for functional studies and gene expression evaluation.

### Functional Assays

Cell cytotoxicity assays were performed using as target NALM-18 cell line (childhood B-cell acute lymphoblastic leukemia) or K562 cell line (erythroleukemia) and as effector cells (mobilized or not) NK cells at different Effector/Target (E/T) cell ratios. Killed cells were evaluated after 4 hours. At the end of the co-culture, the assay was stopped by chilling cells on ice, and Propidium Iodide (PI) was added to each sample immediately before acquisition in order to identify the percentage of target cell lysis, as previously described (29). For each set of experiment, all the acquisitions (5,000 target cells/sample) were performed within 20 min. Statistical analysis was performed using GraphPad Prism.

PMN-MDSC apoptosis was examined using Annexin V-FITC apoptosis detection kit (BD biosciences, Franklin Lakes, NJ, USA). PMN-MDSCs derived from non- and post-mobilized donors were separated and cultured in 24-well plates at a density of 1 × 10^6^ cells/well. Analysis of cell viability and apoptotic status was evaluated at different time points (from 1 to 6 days). PMN-MDSCs were incubated with 5 μl of Annexin V-FITC and 2 μl of PI at room temperature for 15 min. The PMN-MDSC apoptotic rate was measured using a flow cytometer (Cytoflex LX, Beckman Coulter, Brea, CA, USA). Chemotaxis of NK cells was measured by migration through a polycarbonate filter with an 8.0 μm pore size in 24-well trans-well chambers (Sarstedt, Nümbrecht, Germany). The assay medium consisted of Roswell Park Memorial Institute (RPMI) supplemented with 10% fetal bovine serum (FBS) derived from purified PMN-MDSCs non-mobilized and post-mobilization after 24 h of culture. 500 μl of assay medium was added to the lower chamber in the presence or not of CCL4 blocking antibodies (1.2 μg/ml; R&D Systems, Minneapolis, MN, USA). 500 μl of assay medium was used as a control for spontaneous migration. Then, 1 × 10^5^ NK cells were seeded to the upper chamber. After 1 hour of incubation at 37°C, NK cells that migrated to the bottom chamber were harvested and counted by flow cytometry (absolute count).

### RNA Extraction and Real Time PCR Analysis

Total RNA extraction from purified NK cells and PMN-MDSCs was performed with miRNeasy mini kit, combined with on-column DNase I treatment, following the manufacturer’s protocol (Qiagen GmbH, Hilden, Germany). RNA concentration and purity were evaluated by spectrophotometric analysis (Nanodrop 2000; Thermo Fisher Scientific, Wilmington, DE). For gene expression analysis in NK cells and PMN-MDSCs, total RNA was reverse transcribed with random primers using Super Script IV first-strand synthesis system following manufacturer’s instructions (Thermo Fisher Scientific, Wilmington, DE, U.S.A.). Real Time PCR on NK cells was performed by 384-wells TaqMan array microfluidic cards with a custom configuration for the detection of selected genes implicated in NK cell biology (Thermo Fisher Scientific, Wilmington, DE, U.S.A.). Briefly, 200 ng of cDNAs for each sample was mixed with an isovolume of TaqMan Advanced Master Mix 2× and loaded in 384-wells cards (100 ng/channel). Real time PCR on PMN-MDSCs was performed in 20 µl of total volume with TaqMan™ Fast Advanced Master Mix (Applied Biosystems, Foster City, CA, U.S.A). The following TaqMan™ Gene Expression assays were used: MS4A4A (Hs00254780_m1), CLEC7A (Hs01902549_s1), CD177 (Hs00360669_m1), CCL4 (Hs99999148_m1), 18S (Hs99999901_s1). Reactions were carried out on a QuantStudio 12 Flex instrument using thermal PCR cycling conditions suggested by the manufacturer (Applied Biosystems, Foster City, CA, U.S.A). Data analysis was performed on Thermo Fisher Cloud with Design and Analysis New qPCR application (Thermo Fisher Scientific, Wilmington, DE, U.S.A.). PCR array data were analyzed with relative threshold algorithm and normalized using the mean of ACTB and HPRT expression, used as reference genes. Real time PCR on PMN-MDSC samples was analyzed with baseline threshold algorithm and 2^−ΔCt^ method. Data were normalized with 18S housekeeping gene.

### Microarray Analysis

The samples of non-mobilized (n = 5) and mobilized (n = 8) PMN-MDSCs from which we obtained enough amount of high quality total RNA were used as start material for the microarray gene expression analysis. Biotin labeled RNA was generated using the GeneChip 3′ IVT Plus labeling kit (Thermo Fisher Scientific, Wilmington, DE) according to the manufacturer’s instructions. The Biotin labeled RNA was subsequently hybridized to a HT U133 plus 16-array plate (Thermo Fisher Scientific, Wilmington, DE). The hybridization, wash, staining, and scanning procedures were done in a GeneTitan™ Instrument according to the manufacturer’s protocol (cat#: 00-0373; Thermo Fisher Scientific, Wilmington, DE). For labeling, hybridization, washing, staining, and scanning procedures we took advantage of Eurofins Genomics microarray service (Eurofins Genomics; Ebersberg, Germany).

The cel file output was used as input in the Partek Genomics Suite Software (Partek, St. Louis, MO) for generation of Robust Multi-Array Average (RMA) normalized data.

### Bioinformatics and Statistical Analysis

**Figure 1:**
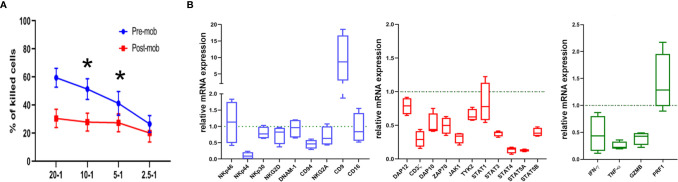
Cytolytic activity and expression of functional genes are altered in NK cells from mobilized grafts. **(A)** Freshly isolated NK cells derived from donors who did (*post-mob*; in red) or did not (*pre-mob*; in blue) undergo mobilization with G-CSF were purified, and their cytolytic activity was assessed using NALM-18 as target cells. Percentages of killed cells ± SEM at different Effector/Target (E/T) ratios are shown. Statistical analysis was performed using Mann–Whitney test (n = 5). *p ≤ 0.05; where not indicated, data were not statistically significant. **(B)** Box and whisker plots representing the expression of the indicated panels of genes (*blue*: cell surface markers; *red*: transduction signal molecules; *green*: effector molecules) measured by real time PCR array in NK cells freshly isolated from apheresis of mobilized donors (n = 4). Values are expressed as fold change with respect to their expression in NK cells from PB of non-mobilized donors, used as control (n = 4).

**Figure 2:**
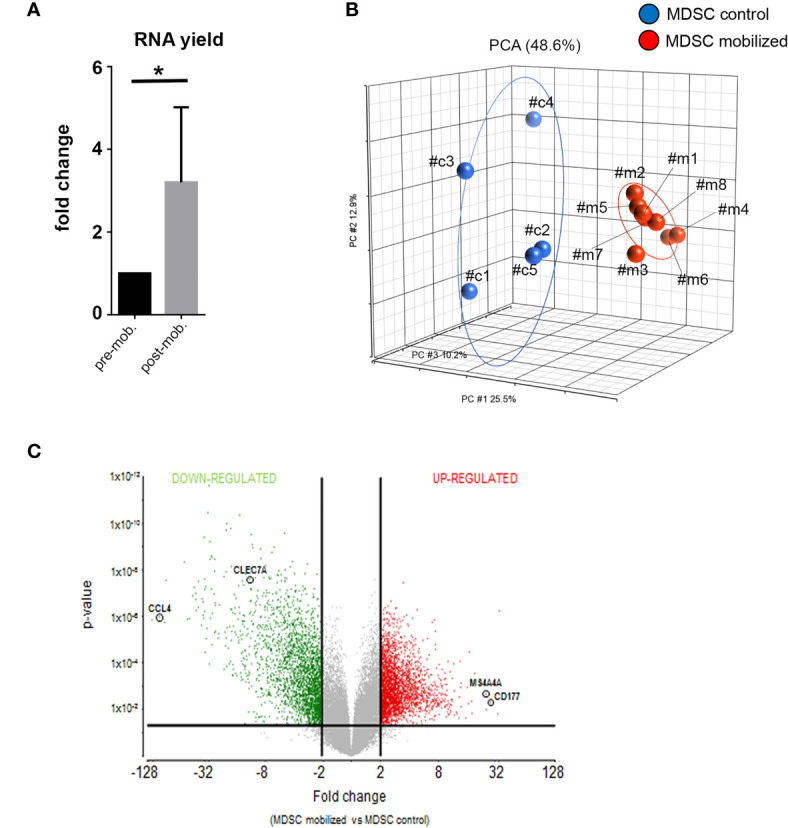
Global gene expression analysis reveal distinct transcriptional profiles in mobilized PMN-MDSCs. **(A)** Total RNA yield obtained from freshly-isolated PB PMN-MDSCs of donors before (pre-mob) and after mobilization (mob). Data are expressed as fold change with respect to non-mobilized samples. Bars indicate SD. *p-value ≤ 0.05 (Student’s t-test). **(B)** Principal Component Analysis (PCA) summarization of microarray data for non-mobilized (MDSC control; blue circles; n = 5) and mobilized (MDSC mobilized; red circles; n = 8) PMN-MDSCs. **(C)** Volcano plot representing DEGs between mobilized and non-mobilized PMN-MDSCs upon ANOVA of microarray data. Vertical lines indicate FC≤-2 and ≥2, and horizontal line indicates p-value ≤ 0.05, representing cut-off lines applied to filter significant DEGs. Probeset significantly up-regulated and down-regulated are highlighted in red or green, respectively. The positions of CCL4, CLEC7A, MS4A4A, and CD177 genes in the volcano plot are highlighted.

**Figure 3:**
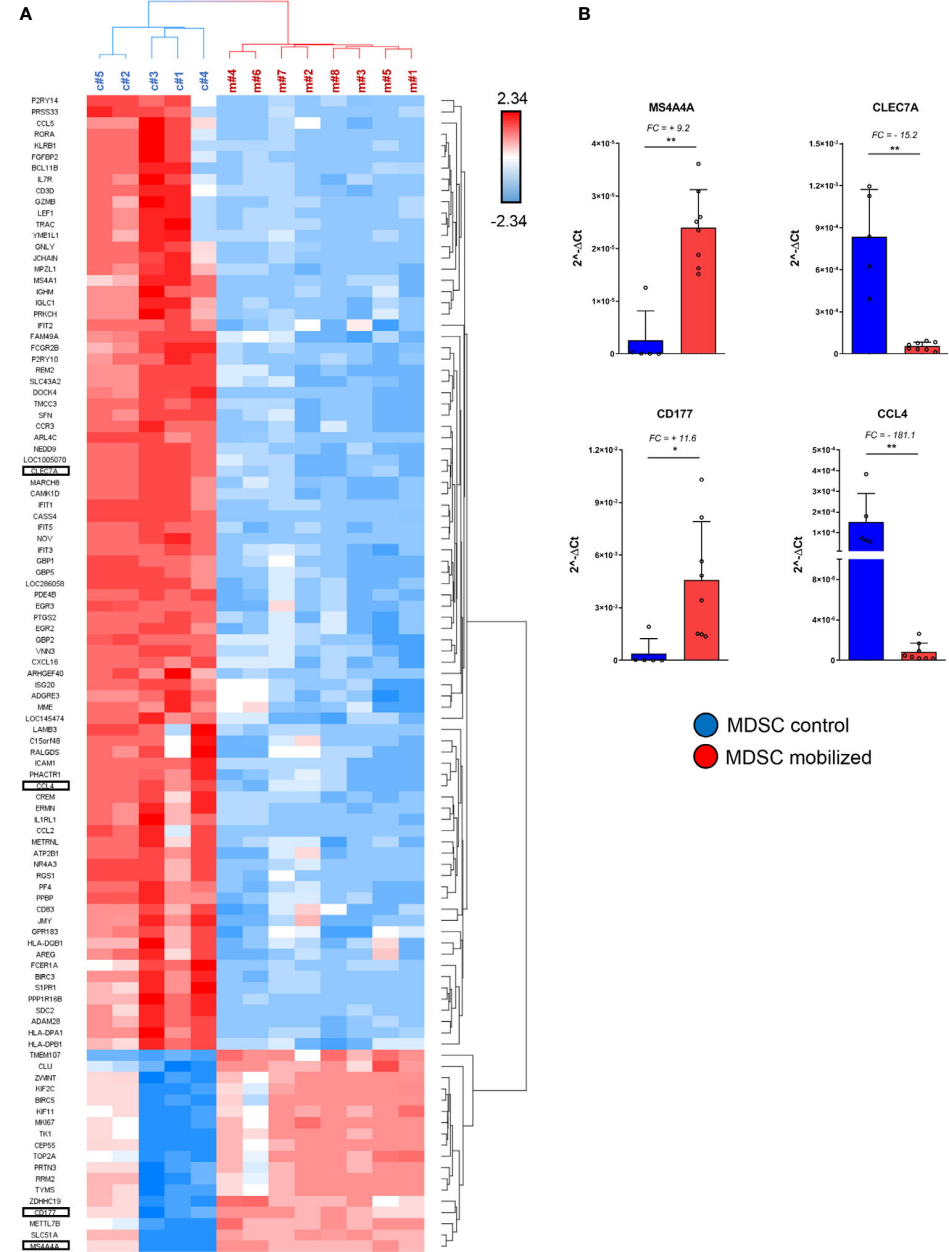
Specific immuno-modulatory genes are differentially expressed in mobilized PMN-MDSCs. **(A)** Heat-map of the top up-regulated and down-regulated genes in PMN-MDSCs, based on the filter FC ≥10 or ≤-10; FDR adjusted p-value ≤ 0.05. Red squares indicate up-regulated genes; blue squares down-regulated genes in mobilized PMN-MDSCs. Each row represents a gene; each column represents individual samples analyzed by microarray (control: c#1–5; mobilized: m#1–8). Specific genes selected for further analysis are highlighted. **(B)** Real time PCR analysis on the samples used for microarray for the indicated genes. Values were calculated with ΔCt method. 18S was used as endogenous control. Bars indicate SD. *p-value ≤ 0.05; **p-value ≤0.01 (Mann–Whitney test).

**Figure 4:**
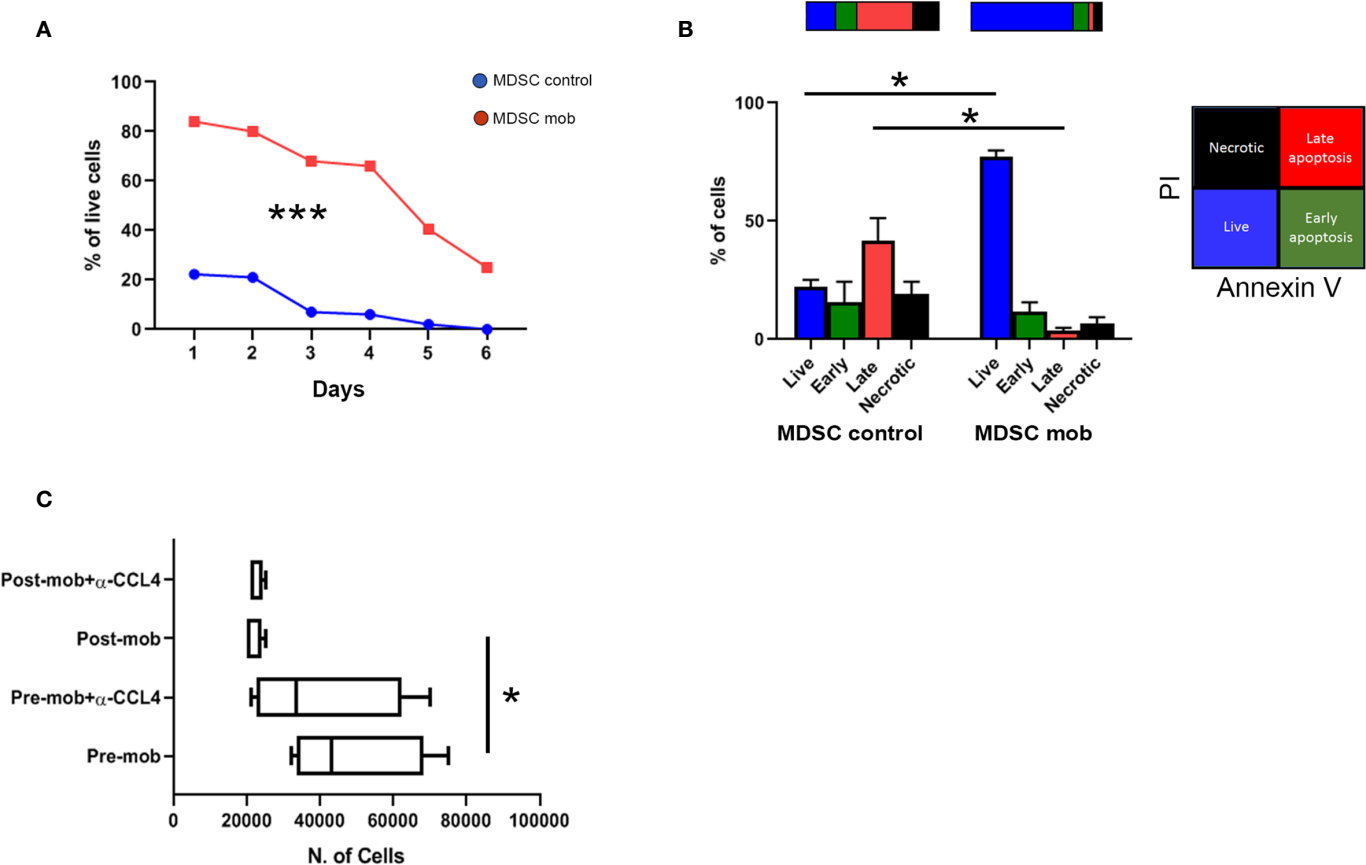
Prolonged survival and altered chemo-attraction of mobilized PMN-MDSCs. **(A)** Percentages of PMN-MDSC live cells (PI^-^) along 6 days of non-mobilized (PMN-MDSCs ctrl, red square line) and mobilized (PMN-MDSCs mob, blue dots line) donors. ***p-value < 0.001 (two-way ANOVA test, n = 3). **(B)** Percentages of live (Annexin V^-^ and PI^-^, blue), early apoptotic (Annexin V^+^ and PI^-^, green), late apoptotic (Annexin^+^ and PI^+^, red), and necrotic (Annexin V^-^ and PI^+^, black) control (left) and mobilized (right) PMN-MDSCs measured by flow cytometry after 24 h of culture. *p-value ≤ 0.05; (Mann–Whitney test, n = 4). **(C)** Numbers of migrated NK cells after 1 h of culturing in conditioned medium derived from pre- and post-mobilized PMN-MDSC, in the presence or not of anti-CCL4 blocking antibody. *p-value ≤ 0.05; (Mann–Whitney test, n = 4).

Data in **Figures 1**, **2A** were expressed as mean ± standard deviation (SD) or ± standard error of the mean (SEM) as indicated. For RNA yield evaluation, statistical significance was calculated using paired Student’s t-test. A p value ≤0.05 was considered statistically significant. DEGs between non-mobilized and mobilized PMN-MDSC groups were calculated by ANOVA one-way test. Lists of DEGs were filtered applying fold change (FC) >2 or <-2 (FC>10 or <-10 in **Figure 3A**) and a p-value ≤0.05 with Benjamini–Hochberg (1995) False Discovery Rate (FDR) correction criteria. PCA, ANOVA test, unsupervised hierarchical clustering in **Figure 3A**, and box plots in **Supplementary Figures 2A, B**, were generated by Partek Genomics Suite (Partek, St. Louis, MO). Enrichment of GO BP terms was performed by GOrilla tool (30) applying a p-value threshold of 10-3, calculated according to the mHG or HG model. Graphical summarization of enriched GO BPs in a semantic treemap was performed by REVIGO allowing medium similarity and applying the simRel score (31). Two-way Anova was used for **Figure 4A**, Mann–Whitney test was used for **Figures 3B**, **4B, C**.

## Results

### NK Cells Present in the Donor Graft Exhibit a Reduced Cytolytic Activity

As previously shown, the HSC mobilization regimen induces accumulation of PMN-MDSCs in the PB of G-CSF-mobilized donors. Moreover, these cells sharply inhibited the effector function of autologous and allogeneic NK cells *in vitro* (28). Based on these results, we verified whether the cytolytic activity of NK cells, isolated from the PB of mobilized donors, was affected by the concomitant presence of high numbers of PMN-MDSCs. Fresh NK cells were isolated from apheresis (post-mobilization) or from PB of unrelated HD, and their ability to kill NALM-18 tumor target cells was assessed. As shown in **Figure 1A**, NK cells isolated from mobilized donors showed a lower cytolytic activity as compared to HD NK cells. As previously demonstrated, PMN-MDSCs are capable, after 48 h of co-culture, to strongly impair the cytolytic activity of NK cells. In order to clearly demonstrate the impact of PMN-MDSCs on NK cell function, we further removed PMN-MDSCs after 48 h from NK/MDSC co-cultures and tested the cytolytic activity of these NK cells after additional 24 h of culture (referred as NK/MDSC 24 h alone; **Supplementary Figure 1**). The inhibitory effect of PMN-MDSCs was reversible, since the cytolytic activity of NK cells could be restored by removing PMN-MDSCs from co-cultures (**Supplementary Figure 1**).

### NK Cells in the Donor Graft Display an Altered Expression of Genes Controlling Immune Effector Functions

To support the functional data, we further investigated whether NK cells of mobilized donors were modified in their gene expression profiles. Thus, we analyzed by PCR array the expression of a wide panel of genes regulating cell function in NK cells purified from mobilized and non-mobilized donors. In particular, we evaluated genes considered important in the control of immune effector function of NK cells. As shown in **Figure 1B**, in comparison to NK cells from HD, “mobilized” NK cells revealed a decrease in the expression of some genes encoding for surface receptors involved in anti-tumor activity including NKp44 and CD94, or signal transduction molecules CD3*ζ*, ZAP70, DAP10, DAP12, or STAT family members, or effector molecules such as IFN-*γ*, TNF-α and GZMB. Interestingly, in NK cells from mobilized donors, we also found a higher expression of the CD9-encoding gene (**Figure 1B**). CD9 up-regulation was reported in NK cells converted into a pro-angiogenic, non-cytotoxic state upon exposure to TGF*β* that, in turn, can be released by PMN-MDSCs as well (32, 33). Taken together, these results suggest that the effector function and the activation status of NK cells in the graft may be influenced by the presence of PMN-MDSCs.

### PMN-MDSCs in the Donor Graft Are Characterized by Distinct Gene Expression Profiles

Next, we verified whether the mobilization regimen induced changes in the molecular properties of PMN-MDSCs present in mobilized donors. First, we compared the frequency of PMN-MDSCs in G-CSF mobilized and non-mobilized donors. In G-CSF mobilized donors PMN-MDSCs were ≈20% while they were <5% non-mobilized donors (28). Their percentages increased to ≈40% in the collection bag (due to their enrichment related to the B- and *αβ* T-cell depletion; data not shown). Notably, RNA yield from freshly isolated PMN-MDSC in the donor graft was significantly higher than that of the same cells isolated from the same pre-mobilized donors (∼three-fold) (**Figure 2A**). The increased RNA content suggested an activated metabolic state in PMN-MDSCs of mobilized grafts. Thus, we investigated whether these cells displayed transcriptional features different from non-mobilized PB PMN-MDSCs. To this purpose, we performed a global gene expression profiling by microarray to compare eight samples of PMN-MDSCs isolated from PB of mobilized donors with a control group represented by five PMN-MDSC samples derived from non-mobilized HD. In the microarray, three out five of the control samples were derived, before mobilization, from the same donors analyzed in the mobilized group (samples *c#1*, *c#,2* and *c#3 versus m#1*, *m#2*, and *m#3* respectively; **Figure 2B**). We used a three-dimensional Principal Component Analysis (PCA) plot to summarize the microarray data. This exploratory analysis showed that all the mobilized PMN-MDSCs samples finely clustered together and were clearly distinguished from non-mobilized PMN-MDSCs samples, indicating that, indeed, the two groups are characterized by distinct transcriptional profiles (**Figure 2B**).

### Specific Pro-Proliferative and Immune-Modulatory Genes Are Differentially Expressed in PMN-MDSCs of Mobilized Donors

We further investigated the differentially expressed genes (DEGs) in mobilized or non-mobilized PMN-MDSCs. To this end, we performed an Analysis of Variance (ANOVA) test, imposing a p-value ≤0.05 and a fold change (FC) ≥|2|. We found 4,625 differentially expressed probe sets displaying robust and statistically significant variations between mobilized and non-mobilized PMN-MDSCs. Among them, 2,589 probe sets were up-regulated and 2,036 down-regulated in mobilized *versus* non-mobilized PMN-MDSCs (**Figure 2C** and **Supplementary Table 1**). Hierarchical clustering based on these significantly deregulated genes also confirmed a clear discrimination between mobilized and non-mobilized PMN-MDSC samples (data not shown). It should be noted that some important genes characterizing the PMN-MDSCs subset such as LOX1, S100A9, GAS6, TGF*β*1 and IL-10 were expressed in both groups with no significantly different expression level, suggesting that mobilized PMN-MDSCs retain some features of typical PMN-MDSCs (**Supplementary Figure 2A**). Remarkably, in mobilized PMN-MDSCs we detected a differential expression of some relevant genes associated with cell proliferation and immunosuppressive function. In particular, MS4A4A (↑), MKI67 (↑), ARG1 (↑), CEACAM1 (↑), FKB5 (↑) genes were up-regulated whereas CCL4 (↓), IL7R (↓) were down-regulated in mobilized PMN-MDSCs (**Supplementary Table 1** and **Figure 3A**).

A heat-map showing the most modulated genes in mobilized PMN-MDSCs is represented in **Figure 3A**. In this analysis, we filtered the list to DEGs with FC ≥ |10| to narrow down to the most deregulated genes. One of the most up-regulated genes in mobilized PMN-MDSCs was CD177 (FC = +27.4) (**Supplementary Figure 2B**), a surface membrane glycoprotein expressed in neutrophils and neutrophil precursors (34). Consistent with the wide up-regulation of other genes involved in MDSC proliferation and activation, this gene is involved in neutrophil proliferation and was found to be up-regulated in human MDSCs upon inflammation (35, 36). Furthermore, the tetraspan molecule MS4A4A was one of the most up-regulated genes in mobilized MDSCs (FC = +24). It has recently been reported that MS4A4A expression is restricted to human mononuclear phagocytes, where MS4A4A co-localizes with the *β*-glucan receptor dectin-1 (CLEC7A), mediates NK cell activation and exert a control of metastases in murine models (37). Our data indicate that Dectin-1/CLEC7A is down-regulated (FC= –11) in mobilized PMN-MDSCs. Since Dectin-1 signaling is crucial for mediating cell-to-cell contact and subsequent NK cell activation, the up-regulation of MS4A4A might have a different role besides inducing NK cell activation *via* MS4A4A-CLEC7A. A sharp increase of MS4A4A expression, usually associated with cells of the monocytic lineage, suggests that PMN-MDSCs may acquire unique molecular characteristics upon G-CSF induced mobilization. Conversely, one of the most down-regulated genes was CCL4, encoding for an inflammatory chemokine functioning as chemoattractant for different leukocytes, including NK cells (**Supplementary Figure 2B**). The above mentioned genes were selected for PCR validation on the same samples used in the microarray. Real time PCR analysis confirmed a striking differential expression of all the genes tested, confirming the reliability of the microarray approach (**Figure 3B**).

### PMN-MDSCs of Mobilized Donors Display Prolonged Survival and Altered Chemo-Attraction Properties on NK Cells

Transcriptome data indicated that mobilized PMN-MDSCs are characterized by an activated phenotype. Thus, we asked whether their distinct gene expression profile was associated with an altered cell survival. To this purpose, mobilized and non-mobilized PMN-MDSCs were cultured *in vitro*, and cell viability was evaluated for 6 days. Whereas non-mobilized PMN-MDSCs underwent rapid cell death upon *in vitro* culturing, mobilized PMN-MDSCs displayed a significantly prolonged survival (**Figure 4A**). Of note, after 24 h of *in vitro* culture, the large majority of mobilized PMN-MDSCs were represented by live cells, whereas their non-mobilized counterpart already showed an extensive fraction of apoptotic cells (**Figure 4B**).

Transcriptional profiles also suggested that mobilized PMN-MDSCs could have different immune properties as compared to the non-mobilized ones. Based on down-regulation of several chemokines including CCL4 (see **Figure 3A** and **Supplementary Table 1**), we tested whether mobilized PMN-MDSCs could exhibit different chemo-attractive properties on NK cells. Supernatants of non-mobilized and mobilized PMN-MDSC cultures displayed significant differences in the capability to attract NK cells in trans-well chambers system. In particular, supernatants of mobilized PMN-MDSCs exhibited a reduced ability to attract NK cells as compared to non-mobilized ones (**Figure 4C**). The use of a blocking antibody against CCL4 chemokine caused a slight decrease of NK cell migration in non-mobilized supernatants, indicating that CCL4 chemokine is involved, at least in part, in this process (**Figure 4C**).

### Biological Processes Associated With Proliferation and Myeloid Activation Are Enriched in Mobilized PMN-MDSCs

**Figure 5:**
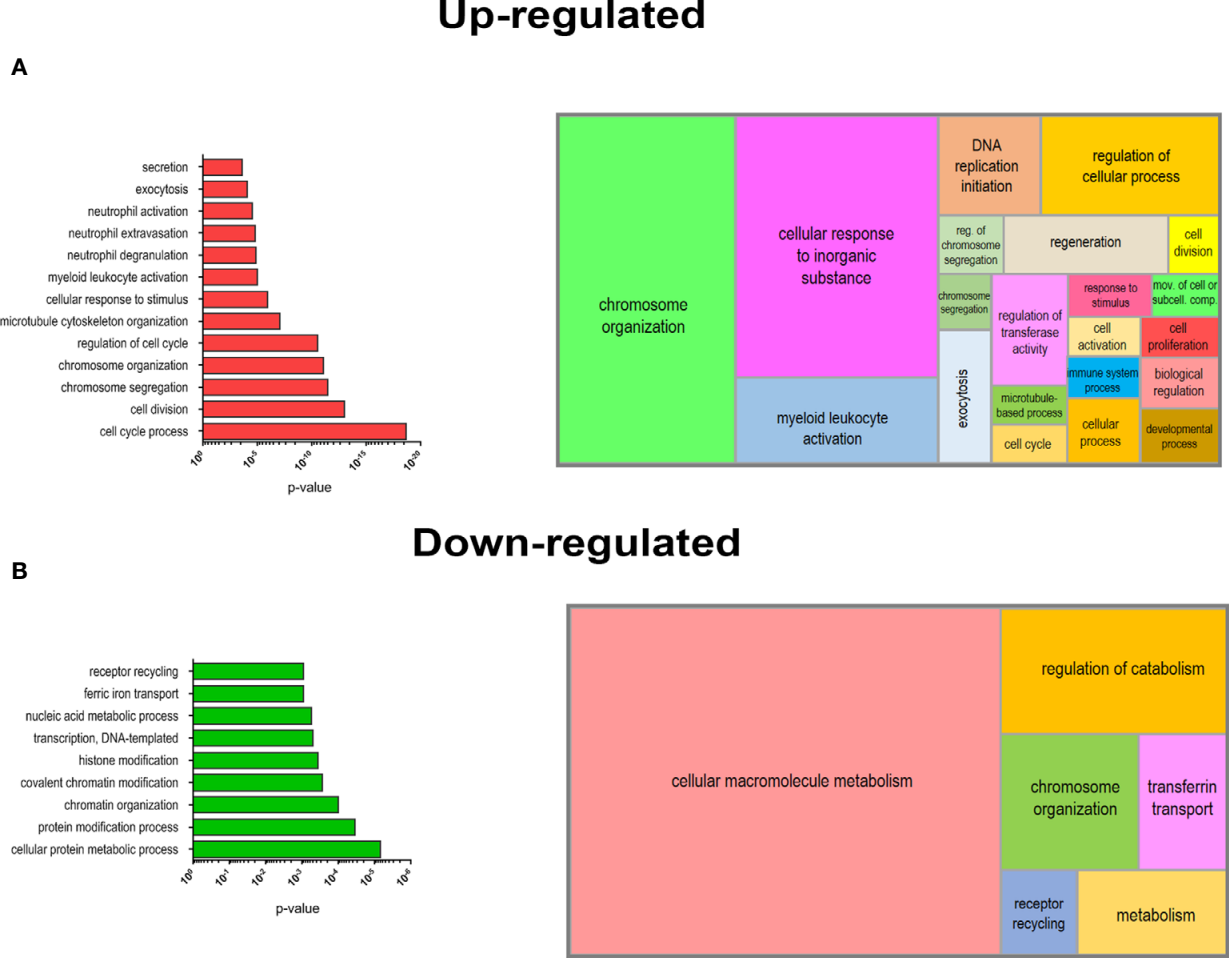
Biological processes (BPs) enrichment in DEGs of mobilized PMN-MDSCs. Selected list of BPs from the GO database enriched for up-regulated **(A)** o r down-regulated **(B)** genes of mobilized PMN-MDSCs (left panels). Histrograms represent −log10 p-value for each enriched BP calculated by GOrilla tool (see *Materials and Methods*). Graphical summarization of the GO BPs significantly enriched in up-regulated and down-regulated gene lists is shown with a semantic clustering performed by REVIGO tool (right panels).

In order to deepen the biological significance of DEGs identified in PMN-MDSCs of mobilized donors, we performed an enrichment analysis of biological processes (BPs) from Gene Ontology (GO) database. We separately analyzed the list of up-regulated (FC ≥2; p value ≤0.05) and down-regulated (FC ≤-2; p value ≤0.05) genes in mobilized MDSCs to find emerging BPs in these groups. A list of BPs significantly over-represented is shown in **Figure 5**, whereas the complete list of enriched BPs in up-regulated and down-regulated genes is reported in **Supplementary Tables 2** and **3**. This analysis clearly highlighted a striking predominance of BP associated with cell proliferation in the up-regulated genes in G-CSF mobilized PMN-MDSCs, including cell cycle process (GO:0022402), chromosome organization (GO:0051276) and segregation (GO:0007059). Another important group of BPs regards the immune defense compartment, in particular, the myeloid cell activation (GO:0002274) and other processes specifically associated with granulocytes, such as neutrophil degranulation (GO:0043312), activation (GO:0042119) and extravasation (GO:0072672) (**Figure 5A**). Conversely, down-regulated genes in mobilized PMN-MDSCs were enriched in some BPs associated with macromolecule metabolism, *e.g.* transcription DNA-templated (GO:0006351) and nucleic acid metabolic process (GO:0090304). Other significantly enriched BPs were associated to covalent chromatin modification (GO:0016569) and histone modification (GO:0016570) (**Figure 5B**). A summarization of the GO BPs significantly enriched in up-regulated and down-regulated gene lists was performed by REVIGO (31) to graphically represent in a semantic tree-map all the BPs identified (**Figure 5**). Collectively, these data suggest that the mobilization regimen with G-CSF induced a transcriptional program promoting cell proliferation and activation of myeloid cells.

## Discussion

In the present paper, we first show that NK cells present in the graft after G-CSF mobilization regimen display a lower cytolytic activity as compared to NK cells from HD. We then performed a detailed gene expression analysis on both NK cells and PMN-MDSCs derived from apheresis after mobilization with G-CSF. Notably, PMN-MDSC and NK cells represent two important cellular components of the *αβ* T cell- and B cell-depleted graft. Since, upon G-CSF-induced mobilization and *αβ* T and B cell-depletion of the graft, PMN-MDSCs are present in high percentages, it was conceivable that they could exert a strong negative impact on NK cell effector function. Notably, mature donor-derived NK cells were shown to contribute to both GvL activity and control of infections at early stages after transplantation, particularly in the haplo-HSCT setting using pure CD34^+^ HSC. However, we found that NK cells present in mobilized graft in *αβ* T and B cell-depleted HSCT setting show a reduced anti-leukemia activity as compared to non-mobilized NK cells. This functional evidence was further supported by transcriptional data, revealing that some molecules involved in NK cell-mediated anti-tumor activity were down-regulated in mobilized samples. Although the reduced cytotoxic activity of NK cells *in vivo* upon G-CSF regimen could reflect also MDSC-independent mechanisms, the high proportion of PMN-MDSCs in the graft raises the intriguing possibility that G-CSF could inhibit donor NK cell cytotoxicity through PMN-MDSC activation and expansion. Indeed, we show that the inhibition of NK cell cytolytic activity *in vitro* is due to the presence of PMN-MDSCs and that their removal results in the restoration of NK cell function. Our data are supported by a previous study in which we showed that PMN-MDSCs present in high concentrations in the *αβ* T and B cell-depleted transplants exert a sharp inhibitory effect *in vitro* on both allogeneic and autologous NK cell function (28).

G-CSF-induced mobilization causes the release in the PB and the expansion of a broad spectrum of hematopoietic cell precursors including PMN-MDSCs. An activated state of these cells was suggested by the increase in the total RNA amount per cell in mobilized donors. Thus, we asked whether PMN-MDSCs in the graft possess peculiar molecular characteristics or whether they are similar to PMN-MDSCs isolated from non-mobilized donors. Interestingly, our comprehensive gene expression profiling by microarray revealed that PMN-MDSCs in the graft showed a peculiar transcriptional profile distinguishing them from non-mobilized PMN-MDSCs. All the mobilized samples finely clustered together and were clearly distinct from non-mobilized ones. Notably, three out of five of the control samples were derived, before mobilization, from the same donors analyzed in the mobilized group (samples *c#1*, *c#2*, and *c#3 versus m#1*, *m#2*, and *m#3* respectively; **Figures 2B** and **3A**), thus further supporting that the variability between the two groups was indeed related to the mobilization regimen. The most prominent difference in mobilized PMN-MDSCs resides in the strong up-regulation of genes promoting DNA replication, cell cycle, and cell division such as the marker of cell proliferation Ki-67 (MKI67), topoisomerase II alpha (TOP2A), cyclin B (CCNB2) or kinesin family member 2 C (KIF2C). Transcriptional data would clearly support the scenario of a rapidly expanding PMN-MDSC population in PB of mobilized donors, in agreement with their high concentration in the graft. Both the high number of PMN-MDSCs in the graft and their possible further expansion after transplantation offer a reasonable explanation for the impaired cytolytic activity of NK cells.

Although there was a pronounced difference in the transcriptome, several key genes associated with PMN-MDSCs such as LOX1, NOS1, GAS6, TGF*β*1, IL10, and S100A9 were not significantly de-regulated in the two compared groups. This finding indicates that PMN-MDSCs isolated from mobilized or non-mobilized donors share a number of similar features and display some common functional properties. On the other hand, the expression of some genes with important immune-regulatory functions was sharply deregulated in mobilized PMN-MDSCs. Among them, we found a strong up-regulation of the tetraspanin MS4A4A. MS4A4A was reported to be a fundamental molecule in tumor-infiltrating macrophages as well as in dectin-1-dependent activation of NK cells in cancer (37). Up-regulation of MS4A4A was reported to be restricted to cells of the macrophage-lineage (37). Of note, we show that such up-regulation also occurs in PMN-MDSCs upon G-CSF induced mobilization. In addition, we found Dectin-1 (CLEC-7A) was significantly down-regulated in these cells. Since Dectin-1 is critical for NK cell-mediated killing of tumor cells (38), it is possible that MS4A4A may not be functional in mobilized PMN-MDSCs. Interestingly, an increased expression of MS4A4A was also observed in circulating MDSCs after surgical sepsis (35), suggesting that pro-inflammatory stimuli could up-regulate this molecule in MDSCs. Further studies are clearly needed to clarify the functional significance of MS4A4A up-regulation in these cells.

We also observed a striking increase of several other genes encoding for transmembrane proteins, suggesting that the membrane surface composition of mobilized PMN-MDSCs may be significantly different from that of their non-mobilized counterpart. Among these genes, the one encoding the transmembrane protein 107 (TMEM107) is the most up-regulated in mobilized PMN-MDSCs. This molecule plays a critical role in ciliated cells, while to date, their potential functional role in blood cells remains to be defined. CD177 is another of the most up-regulated genes in mobilized PMN-MDSCs. CD177 has been associated with subpopulations of immature developing human neutrophils and it might be involved in their migration (34). Altered migration properties of these cells were also suggested by the up-regulation of other genes such as integrin alpha 9 (ITGA9). Of note, genes regulating neutrophil extravasation were up-regulated in mobilized PMN-MDSCs. Thus, the migration properties and the ability to extravasate may greatly differ in these cells as compared to other mature neutrophil populations.

Regarding the group of down-regulated genes, enrichment analysis underlines an emerging and strong over-representation of genes involved in transcriptional regulation and chromatin remodeling, further supporting the occurrence of a wide alteration of the metabolic state in mobilized MDSCs as well as of their prolonged survival, possibly associated with a trained immunity process. Metabolic rewiring could be explained as the response of PMN-MDSCs to the environment upon G-CSF stimulus, leading to their activation and expansion. As described above, these changes could also affect immune properties of PMN-MDSCs in the graft modulating their capability to attract NK cells. In this regard, it is also worth mentioning the sharp down-regulation occurring in the CCL4 gene expression. This chemokine is produced by neutrophils and is an important chemo-attractant for NK cells and other immune cells.

A deeper comprehension of the molecular characteristics and functional effects of PMN-MDSCs could highlight the impact of these cells in the HSCT.

Circulating and tumor-infiltrating PMN-MDSCs have a short *in vivo* lifespan, whereas during inflammation the half-life of PMN-MDSCs may be significantly prolonged (39). The persistence of mobilized PMN-MDSCs in transplanted recipient patients and their possible durable effects on NK cells is an interesting, unanswered, question. The evidence that mobilized PMN-MDSCs display an increased survival *in vitro* as compared to non-mobilized PMN-MDSCs suggests they may persist for longer time intervals *in vivo*. However, further investigation is needed to directly address this question.

For example, removal or inactivation of PMN-MDSCs could represent a promising strategy to restore/improve the NK-mediated GvL activity and to control viral infections. On the other hand, PMN-MDSCs could also exert a control on GvHD due to their potent immunosuppressive effect on T cells.

The immunologic and bioinformatics-based analyses of PMN-MDSC cell function in response to donor treatment with G-CSF may be useful for better understanding their interaction with donor effector cells involved in GvL, while a better comprehension of the molecular mechanisms involved in the function of PMN-MDSCs may allow for exploiting or inactivating these cells not only in HSCT but also in other pathological conditions.

## Data Availability Statement

The datasets presented in this study can be found in online repositories. The names of the repository/repositories and accession number(s) can be found below: https://www.ncbi.nlm.nih.gov/geo/, GSE159069.

## Ethics Statement

The studies involving human participants were reviewed and approved by the Bambino Gesù Children’s Hospital (Rome, Italy) ethics committees (protocol number 1724/2018). The patients/participants provided their written informed consent to participate in this study.

## Author Contributions

AP designed and performed the research, interpreted the data, and wrote the article. FB performed the experiment and analyzed the data. GLP, PM, and MA provided samples and discussed the data. LQ and LM interpreted the data and revised the manuscript. PV and NT designed the research, interpreted the data, and wrote the article. All authors contributed to the article and approved the submitted version.

## Funding

This work was supported by grants awarded by Associazione Italiana per la Ricerca sul Cancro (AIRC)-Special Program Metastatic disease: the key unmet need in Oncology 5X1000 2018 Id. 21147 (LM), AIRC IG 2017 Id. 19920 (LM); RC-2021 OPBG (LM, PV). LQ has received funding from AIRC and from the European Union’s Horizon 2020 research and innovation program under the Marie Skłodowska-Curie grant agreement no 800924. NT and FB are supported by an AIRC fellowship for Italy.

## Conflict of Interest

The authors declare that the research was conducted in the absence of any commercial or financial relationships that could be construed as a potential conflict of interest.
