# Supplementary material for: NK Cells and PMN-MDSCs in the Graft From G-CSF Mobilized Haploidentical Donors Display Distinct Gene Expression Profiles From Those of the Non-Mobilized Counterpart
Source: Front Immunol. 2021 Apr 27;12:657329. doi: 10.3389/fimmu.2021.657329 (PMC8111072; doi:10.3389/fimmu.2021.657329)
Supplement: Supplementary file 1 [file DataSheet_1.docx]

**Figure S1.** One representative experiment using activated HD-NK cells cultured: alone (NK, black dots); with PMN-MDSC for 48h (NK/MDSC, white square); with PMN-MDSC for 48h and subsequently, upon removal of PMN-MDSC, cultured alone for additional 24h and used (NK/MDSC+24h alone, black triangle). Percentages of killed K562 target cells at different effector/target ratios are shown.

**Figure S2.** Several genes associated to PMN-MDSC phenotype display (a) similar and (b) differential expression in mobilized and non-mobilized PMN-MDSC. Box and whiskers plots showing expression signals of the indicated genes obtained from microarray analysis. Values in Y-axis represent log2 transformed normalized signals for non-mobilized control (*in blue*) and mobilized (*in red*) PMN-MDSC. Dots represent individual samples analyzed in microarray. The following probeset ID from HT U133 plus Affymetrix chip were used for dot plot visualization of differentially expressed genes: MS4A4A: 219607_PM_s_at; CLEC7A: 1554406_PM_a_at; CD177: 219669_PM_at; CCL4: 204103_PM_at.
