# Supplementary figures and images for: NK Cells and PMN-MDSCs in the Graft From G-CSF Mobilized Haploidentical Donors Display Distinct Gene Expression Profiles From Those of the Non-Mobilized Counterpart
Source: Front Immunol. 2021 Apr 27;12:657329. doi: 10.3389/fimmu.2021.657329 (PMC8111072; doi:10.3389/fimmu.2021.657329)

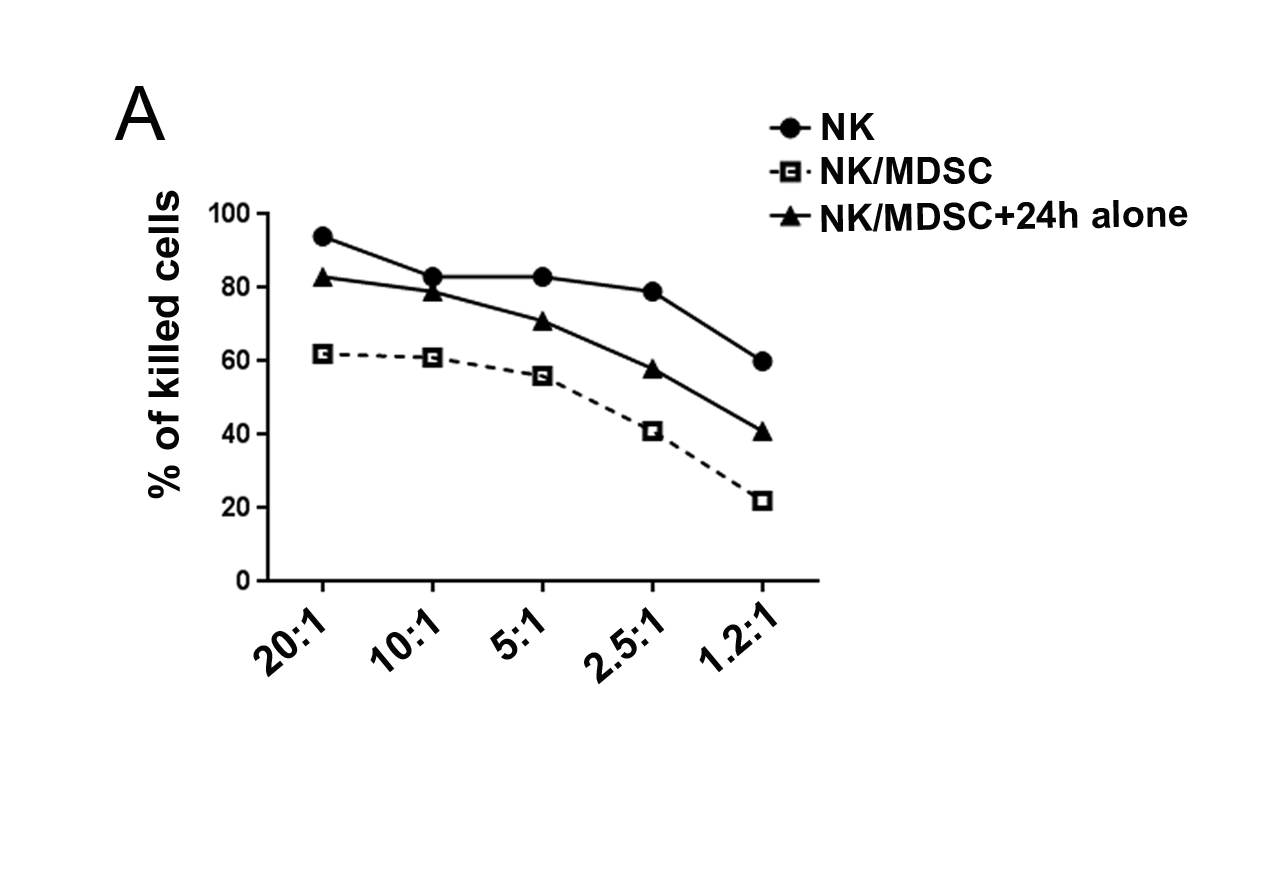

Supplement: Supplementary file 2 [file Image_1.tif]

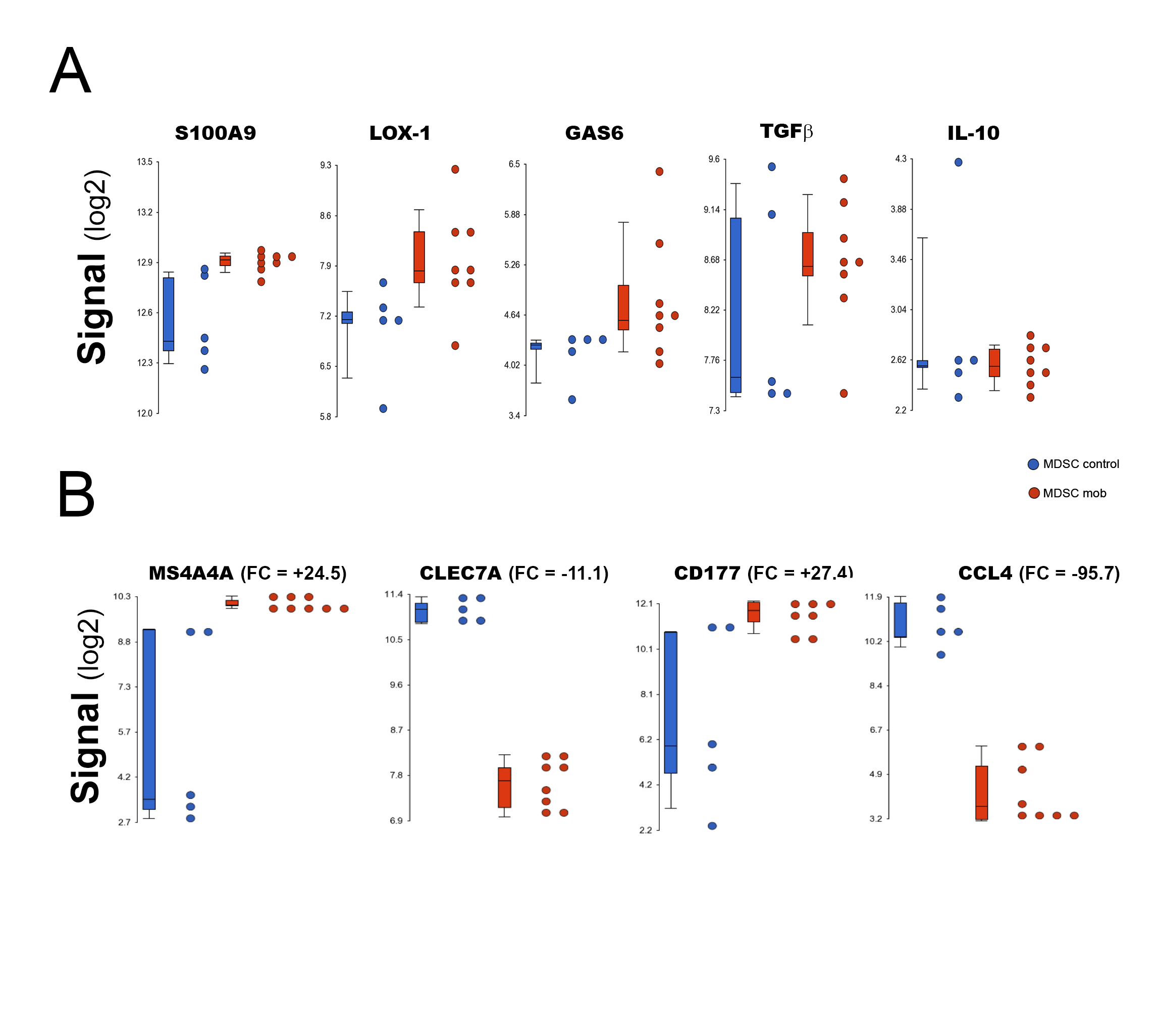

Supplement: Supplementary file 3 [file Image_2.tif]
